# Bioprocess optimization for enhanced xylitol synthesis by new isolate *Meyerozyma caribbica* CP02 using rice straw

**DOI:** 10.1186/s13068-024-02475-8

**Published:** 2024-02-24

**Authors:** Saumya Singh, Shailendra Kumar Arya, Meena Krishania

**Affiliations:** 1 Department of Biotechnology, University Institute of Engineering and Technology, Panjab University, Chandigarh, India; 2 Center of Innovative and Applied Bioprocessing (DBT-CIAB), Sector-81 (Knowledge City), Mohali, 140306 India

**Keywords:** Rice straw, Lignocellulose, Xylitol, Bioreactor, Fermentation, Pretreatment

## Abstract

The present work models the fermentation process parameters of the newly isolated, *Meyerozyma caribbica* CP02 for enhanced xylitol production and its fermentability study on rice straw hydrolysate. The study examined the impact of each of the process variables by one variable at a time optimization followed by statistical validation. Temperature of 32 °C, pH of 3.5, agitation of 200 rpm, 1.5% (v/v) inoculum, 80 gL^−1^ initial xylose was optimized. Subsequently, a sequential two-stage agitation approach was adopted for fermentation. At these optimized conditions, xylitol yield of 0.77 gg^−1^ and 0.64 gg^−1^ was achieved using media containing commercial and rice straw derived xylose, respectively. For scale up, in 3L batch bioreactor, the highest xylitol yield (0.63 gg^−1^) was attained at 72 h with rice straw hydrolysate media containing initial xylose (59.48 ± 0.82 gL^−1^) along with inhibitors (1.55 ± 0.10 gL^−1^ aliphatic acids, 0.0.048 ± 0.11 gL^−1^ furans, 0.64 ± 0.23 gL^−1^ total phenols). The results imply that even under circumstances characterized by an acidic pH and elevated initial xylose level, *M. caribbica* CP02, as an isolate, displays robustness and shows favorable fermentability of rice straw hydrolysate. Therefore, isolate CP02 has potential to be used in bio-refineries for high yield xylitol production with minimal hydrolysate processing requirements.

**Graphical Abstract:**

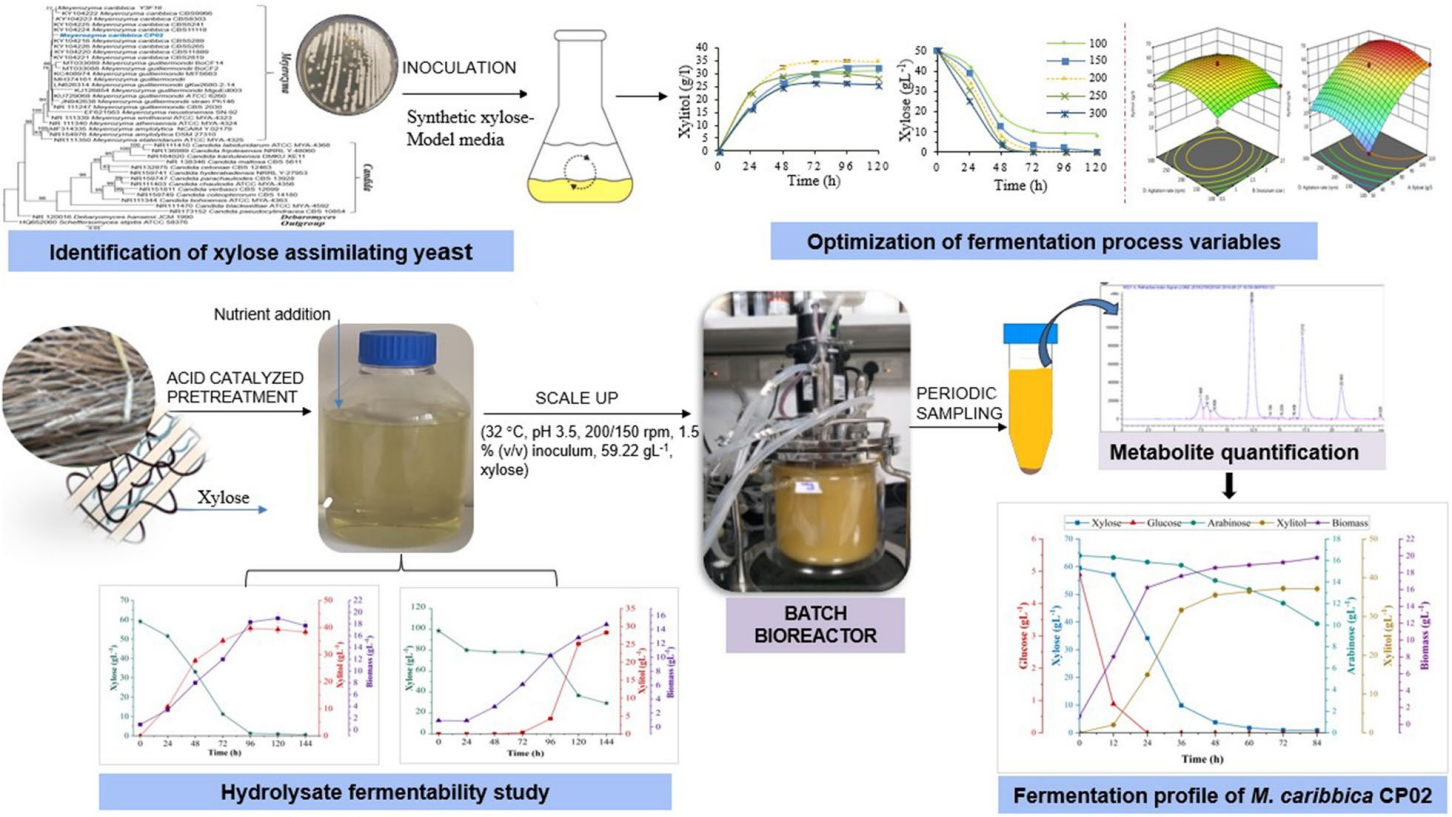

**Supplementary Information:**

The online version contains supplementary material available at 10.1186/s13068-024-02475-8.

## Introduction

Rice is a staple food crop for most of the world's population, with a global production of 730 MT/yr and a massive generation of rice straw (RS) as a subsequent waste by-product. There is currently no sustainable method for managing the large amount of RS produced, 741–1111 MT/yr (290 kg RS/ton of milled rice). Most of it is either left on the ground or burned on site, which has several ecological, health, and environmental consequences [1, 2]. Moreover, straw burning culminates in the accumulation of atmospheric contaminants, such as carbon monoxide, carbon dioxide, volatile organic compounds, oxides of sulphur and, polycyclic aromatic hydrocarbons which deteriorates the air quality and crop yield further [3]. Hence, there is an urgent need to develop large-scale conversion technologies to valorize RS for the development of commercially relevant value-added products such as xylitol. The biotransformation of xylose into xylitol from hemicellulose of lignocellulosic biomass such as rice straw is also of interest to modern biorefineries, as it can be processed into a variety of bio-based chemical products after the cellulosic fraction has been used for ethanol production. According to the US Department of Energy, xylitol is one of the top twelve carbohydrate-derived chemicals with the potential to be a co-product of plant biomass-led bio-refinery [4] for instance, with lactic acid [5] and ethanol [6] which is one of the most advanced productions from lignocellulosic biomass. Xylitol has been approved for use in food products in over 50 countries for dietary purposes, particularly for food, pharmaceutical and odontological applications. Additionally, xylitol also possesses good stabilizing, moisturizing and cryoprotectant properties and has applicability in cosmetics and polymer industries too [7]. Currently, the annual market sale of xylitol is $823.6 million, with a projected increase to $1.37 billion by 2025 [8]. Commercially, xylitol production entails catalytic hydrogenation of D-xylose under extremely high temperature and pressure conditions, making the entire process very expensive and energy intensive. The biotechnological route allows for cheaper xylitol production while also allowing for the sustainable use of excess RS. Factors influencing microbial xylitol production include initial xylose concentration, the presence of other monosaccharides (primarily glucose and arabinose), process parameters (pH, temperature, oxygen supply, etc.), and the presence of microbial inhibitors (organic acids, furans, and phenolics), among others. Oxygen is required for yeasts to metabolize xylose, as it is linked to ATP formation, co-enzyme regeneration, and sugar transport throughout the oxidative phosphorylation pathway [9, 10]. On one hand, high shaking speed promotes cell biomass growth, while on the other it is detrimental to xylitol concentration as the latter is only accumulated under low oxygen conditions [11]. This issue may be solved by achieving a balance between biomass growth and xylitol accumulation during the elongated stationary phase under microaerobic conditions. Furthermore, RS must be pretreated before it can be used for yeast-mediated xylitol production, and the composition of RS hydrolysate (RSH) directly influences the microbe's fermentation performance, cell biomass formation, and, ultimately, xylitol production [12]. Levulinic and formic acids are produced during pretreatment as a result of furan decomposition, whereas acetic acid is produced by the release of acetyl groups from hemicelluloses. HMF and furfural are formed because of hexose and pentose degradation respectively, in addition to other phenolic compounds produced by lignin degradation. All these compounds act cumulatively to either deter or decrease xylitol production by microbes [13, 14]. Additionally, due to the inherently low xylose amount present in RS, 15.10–19.0% (w/w) as compared to other lignocellulosic biomasses such as corn cob, 28.0–31.1% (w/w), and sugarcane bagasse, 21.80–27.0% (w/w), it needs to be extensively treated and concentrated to achieve the desirable xylose level, which is accompanied by a rise in the total inhibitor amount as well [15, 16, 16], Parades et al. [15, 17]. To facilitate low-cost fermentation operations, it is desirable to isolate novel yeast strains with good xylose to xylitol conversion ability and inhibitor tolerance [18, 19]. *Candida* sp*.* is the main xylitol producer; however, some of its species are also considered opportunistic pathogens, limiting its overall applicability, especially in food-led industries [20]. In the present bioconversion study, a two-step agitation and aeration pattern for xylitol synthesis improvement was investigated, beginning with a shake flask, and progressing to a 3L batch bioreactor, for aggrandized xylitol synthesis by optimizing all operational parameters. This work also studied the effects of compositional influence of RS hydrolysate at different xylose concentrations on the behaviour of the newly isolated *M. caribbica*. The robustness and potential of wild isolate *M. caribbica* CP02 has also been validated, which produced remarkable xylitol yield from minimally processed RS hydrolysate under highly acidic condition together with elevated initial xylose and total inhibitor concentration. Considering, the effectiveness of *M. caribbica* CP02 at assimilating xylose, prepares the ground for its potential application in an integrated biorefinery environment driven by biomass.

## Materials and methods

All the components used for media formulation were of analytical grade procured from Hi-media, India, and standards from Sigma Aldrich.

### Screening of xylose-utilizing microorganisms for xylitol production

1 g of soil samples collected from RS dumping yard, garden, sugarcane field, cow dung and decaying wood, were inoculated each in 500 ml Erlenmeyer flasks containing 100 ml sterile YPX media (% w/v): 0.25 yeast extract, 0.50 peptone, 3 xylose (pH 5.5) and incubated at 32 °C for 96 h in a rotary shaker (Climo-Shaker ISF1-X, Basel, Switzerland) at 150 rpm. 100 µl of broth from each of the inoculated flasks was serially diluted and spread on nutrient agar plates containing 1% (w/v) xylose as the principal carbon source and incubated overnight at 32 °C. A loopful of the resulting distinct microbial colonies was taken from each plate and inoculated in 100 ml flasks with 40 ml of YPX media and incubated at 150 rpm for 96 h. The isolate CP02 was chosen for further research based on its xylitol production efficiency, and identification was performed at IMTECH, Chandigarh.

### Morphological analysis and identification of CP02

Inverted microscopy was used to determine the colony and cell morphology of the selected isolate. Phylogenetic analysis was performed using the sequences that were downloaded based on Blast search similarity of ITS and 26S rRNA gene (LSU) regions and recently published data [21, 22]. Thermo Scientific yeast genomic DNA purification kit was used to extract DNA and primer-assisted gene amplification was then performed. The following parameters were used for the PCR amplification: a 5 min initial denaturation at 95 °C, 35 cycles of denaturation at 95 °C for 90 s, primer annealing for ITS (ITS1 and ITS4) and LSU (LR0R and LR5) at 52 °C, primer extension at 72 °C for 1 min, and a final extension step of 10 min at 72 °C. A Gene Jet PCR product purification kit was used to purify the PCR amplicons, followed by sequencing [23, 24]. The multiple sequence alignment for individual gene regions was carried out online at the MAFFT server (http://mafft.cbrc.jp/alignment/server/) [25], and alignments were manually corrected using BioEdit [26]. Maximum likelihood trees under the GTR + GAMMAI (GTR substitution model with gamma- distributed rate heterogeneity) model with 1000 bootstrap replicates was constructed using RAxML-HPC2 on XSEDE (8.2.8) [27, 28] at the CIPRES Science Gateway platform [29].

### Determination of factors affecting xylitol production – OVAT

For preliminary analysis of the operational factors determining fermentation, one variable at a time (OVAT) approach was used. In a 100 mL flask, 40 mL production medium containing (gL^−1^) 50.0 xylose, 10 yeast extract, 10 peptone, 1 MgSO_4_.2H_2_O, 5 CaCl_2_.7H_2_O, 5 KH_2_PO_4_, pH 5.5 was inoculated with 1.0% (v/v) of a 18 h old seed culture and incubated at 32 °C for up to 120 h. The effect of incubation temperature on xylitol yield was studied by carrying out the fermentation from 28 to 36 ^∘^C. The influence of media pH was studied at several pH values ranging from 3.0 to 7.0 at above mentioned conditions. The effect of rate of agitation was analyzed by varying the rpm from 100 to 300. Likewise, the influence of inoculum size was studied by varying the inoculum concentration from 0.5 to 9.0% (v/v). The effect of initial substrate concentration was analyzed in the range of 50 to 100 gL^−1^ xylose.

### Experimental RSM design for optimization of process parameters

Based on the optimal operational values of process variables attained, the Box Behnken Design of Response surface methodology (RSM) was used to statistically optimize the important fermentation process parameters further. Also, the interaction of these variables on xylitol production was studied. Based on this, a more specific range of variables in RSM was considered and coded as A (coded: 50–110 gL^−1^ xylose), B (coded: 0.5–2.5% (v/v) inoculum, C (coded: 2.5–4.5 pH), and D (coded: 100–300 rpm agitation rate). All experiments were performed at 32 °C as determined previously by OVAT. Regression analysis was performed using the statistical software Design Expert 11.1.2.0 (Stat Ease, Minneapolis, USA) to determine the cumulative influence of independent variables on the response. A set of 30 runs with 6 central points was performed discretely and in randomized manner (Table 1T). The significance of every model value was established with ANOVA.

**Table 1:** Chemical composition of pretreated RSH before and after detoxification, followed by concentration

Pretreat-ment	Sugars (gL^−1^)			Aliphatic acids (gL^−1^)			Furans (gL^−1^)		Total phenols (gL^−1^)
	Glucose	Xylose	Arabinose	Acetic acid	Formic acid	Levulinic acid	5-HMF	Furfural	
UTRSH^#^	1.0 ± 0.26^d^	14.91 ± 0.53^d^	3.60 ± 0.79^d^	1.5 ± 0.13^b^	0.28 ± 0.08^a^	0.04 ± 0.01^a^	0.029 ± 0.017^b^	0.132 ± 0.002^d^	0.98 ± 0.007^d^
TRSH	0.93 ± 0.06^d^	12.47 ± 0.17^e^	2.34 ± 0.05^d^	0.89 ± 0.02^c^	0.06 ± 0.01^a^	0.0001 ± 0.0001^b^	0.0003 ± 0.0001^a^	0.005 ± 0.002^e^	0.19 ± 0.04^e^
RSH*1	3.70 ± 0.54^c^	59.22 ± 1.0^c^	12.04 ± 1.00^c^	1.43 ± 0.05^b^	0.09 ± 0.03^a^	0.02 ± 0.01^a^	0.015 ± 0.002^a^	0.034 ± 0.002^c^	0.64 ± 0.026^c^
RSH2	5.53 ± 0.3^b^	78.62 ± 0.71^b^	20.30 ± 0.33^b^	2.06 ± 0.16^a^	0.11 ± 0.07^a^	0.03 ± 0.02^a^	0.018 ± 0.001^a^	0.044 ± 0.002^b^	1.80 ± 0.036^b^
RSH3	7.82 ± 0.65^a^	98.73 ± 0.93^a^	27.45 ± 0.65^a^	2.17 ± 0.20^a^	0.25 ± 0.03^a^	0.05 ± 0.02^a^	0.022 ± 0.013^a^	0.059 ± 0.002^a^	2.47 ± 0.052^a^

^#^UTRSH; Rice straw hydrolysate obtained after steam pretreatment; TRSH; activated charcoal treated hydrolysate; * RSH; Detoxified and vacuum concentrated rice straw hydrolysate consisting of initial xylose concentration adjusted at, 1) 59.22 ± 1.00, 2) 78.62 ± 0.71 and, 3) 98.73 ± 0.93 gL^−1^

Depicted values are the averages ± standard deviations of the outcomes of three independent replicate experiments. Significant differences were evident in values with different letters in the same column (P < 0.05)

### Study of two stage agitation pattern for improved xylitol production

Next a sequential variation in agitation was investigated as an approach to further increase xylitol yield by the isolate CP02. The experiment was performed with a higher agitation in the first phase which enables a better oxygenation followed by lowering the agitation in second phase of fermentation process for xylitol accumulation under oxygen-deficient condition. Fermentation was carried out at optimized conditions in 500 ml shake flask containing 100 ml production media (80 gL^−1^, initial xylose) at 32 °C for 96 h by varying the rpm after time interval of 34 h, as optimized previously (data not included) in various combinations such as 250/200, 250/100, 250/150, 200/150 and 200/100 [30]. The flask shaking constantly at 200 rpm, throughout the entire incubation period was taken as control.

### Xylose rich RS hydrolysate preparation

RS was procured from local farms in Mohali region of Punjab, India. The RS was subjected to dilute acid catalyzed steam pretreatment using 1.5% v/v H_2_SO_4_ for 30 min according to the method proposed by Singh et al. [31] and modified accordingly. After bringing the acidic RSH (rice straw hydrolysate) to pH 3.5 with Ba(OH)_2_, it was centrifuged for 25 min to remove the insoluble salt, Ba(SO_4_)_2_. The supernatant was mixed with 2.0% w/v activated charcoal powder at 60 °C for 1 h before being removed by vacuum filtration through a 0.45 µm cellulose acetate membrane filter. The detoxified hemicellulosic hydrolysate liquor was subdivided into three parts and concentrated under vacuum using a rotary evaporator (Rotavapor RV.10, IKA Staufen, Germany) to achieve final xylose levels of 60, 80, and 100 gL^−1^. The concentration of monosaccharides, aliphatic acids, and furans in the detoxified and concentrated hydrolysate was estimated using the collected aliquots.

### Shake-flask study of detoxified and concentrated RSH for xylitol production

500 mL flasks containing 100 mL RSH set to different xylose levels were prepared according to standardized media conditions and supplemented with gL^−1^: 7.5 yeast extract, 1 MgSO_4_.7H_2_O, 0.5 CaCl_2_, 0.5 KH_2_PO_4_, pH 3.5 and sterilized at 110 °C for 15 min (see Additional file 1: Fig. S1). The Erlenmeyer flasks were inoculated with 1.5% (v/v), 18 h old inoculum culture and incubated at 32 °C and 200 rpm for 34 h before switching to 150 rpm for the remaining period.

### Xylitol production from RSH in a 3L batch bioreactor

Batch process for xylitol production was performed in a 3L bench top Bioreactor (Bio-Spin series, Bio-Age Equipment and services, Mohali, India) with 2.5 L working volume, equipped with pH probe (Broadley James, USA), dissolved oxygen sensing probe (Broadley James, USA) and a temperature sensor. As previously described, detoxified RSH medium with xylose level adjusted to approximately 60 gL^−1^ was used as a fermentation medium upon nutrient supplementation and steam sterilization. Prior to sterilization, the pH of the media was adjusted to 3.5 with 0.1N HCl. The inoculum size was 1.5% v/v and the temperature was held constant at 32 °C. As an anti-foaming agent, 1.0% (v/v) polypropylene glycol was used. For the first 24 h, the airflow was kept at 1.2 Lmin^−1^ and 200 rpm agitation, then reduced to 0.6 Lmin^−1^ and 150 rpm respectively, for the remaining time. Samples were taken on a regular basis for metabolite and cell biomass quantification. "LABFIT" tool (V 7.2.50, Campina Grande, Brazil) and a non-linear regression model were used to study the fermentation kinetics. The logistic model's predictions and the experimental production rates were compared using the 95% confidence interval (CI).

### Analytical methods

Microbial cell biomass was evaluated by computing absorbance at 600 nm in spectrophotometer (UV-1900i Shimadzu Corporation Kyoto, Japan). Morphological analysis of the microbial cell and colony characteristics was performed by Inverted microscope (Nikon H600L, Nikon, Japan) equipped with Nikon’s Digital Sight TS2-S-SM and Scanning electron microscope (Jeol, JCM-6000 benchtop SEM, Tokyo, Japan). Extracellular metabolite (acetic acid, formic acid, levulinic acid, glucose, xylose, arabinose, xylitol, arabitol, HMF and furfural) quantification was conducted by HPLC (Agilent, HiPlex, Santa Clara, California, USA) equipped with a Hi-Plex H column (300 × 7.7 mm, Agilent) coupled to a Refractive index detector. Five mM H_2_SO_4_ was used as the mobile phase at a flow rate of 0.6 mL/min. Column temperature was maintained at 60 °C and that of detector at 55 °C. The concentration of total phenols was estimated by Folins Method [32].

### Estimation of fermentation parameters

The yield of xylitol (Y_xylitol/S_; g/g) was computed by angular coefficient from a linear regression of plot between xylitol concentration, P (gL^−1^) and xylose concentration, S (gL^−1^). Xylitol productivity, Q_p,_ (gL^−1^ h^−1^) was calculated by the highest xylitol production (gL.^−1^) and fermentation time (h), using formula: ${\text{Qp}}=\frac{{\text{g}}/{\text{L}}}{{\text{h}}}$. The efficiency of xylitol conversion (η_xylitol_) was calculated by formula [18]: $\mathrm{\eta_{xylitol }}= \frac{{{\text{Y}}}_{\frac{{\text{P}}}{\mathrm{S }({\text{xylitol}})}}}{{{\text{Y}}}_{\frac{{\text{P}}}{\mathrm{S }(\mathrm{theoretical yield})}}}$

Where, the xylitol yield coefficient is 0.90 g/g of xylose, estimated theoretically for the redox-balanced semi-aerobic production of xylitol in yeasts possessing only NADPH dependent XR activity by the equation:

$${6}0{\text{C}}_{5} {\text{H}}_{{1}0} {\text{O}}_{5} + {\text{ 12ADP }} + {\text{ 12 Pi }} + {\text{ 12 H}}_{2} {\text{O}} + {\text{3O}}_{2} \to {\text{ 54C}}_{5} {\text{H}}_{{12}} {\text{O}}_{5} + {\text{ 12ATP }} + { 3}0{\text{CO}}_{2}$$

[33].

For biomass growth: ${1}.0{\text{95CH}}_{2} {\text{O }} + \, 0.{2}00{\text{NH}}_{3} \to {\text{CH}}_{{1}.{79}} {\text{O}}_{0.{5}0} {\text{N}}_{0.{2}0} + \, 0.{4}0{\text{5H}}_{2} {\text{O}}$ [34].

### Statistical analysis

All experiments were performed in triplicates and results were calculated as the mean ± standard deviation (SD). To assess the significant differences between results, one-way ANOVA followed by Tuckey HSD post hoc test was applied at a probability level (P) ≤ 0.05 using GraphPad Prism software, version 10.

## Results and discussions

### Isolation and identification of xylitol producing yeast strain

**Fig. 1:**
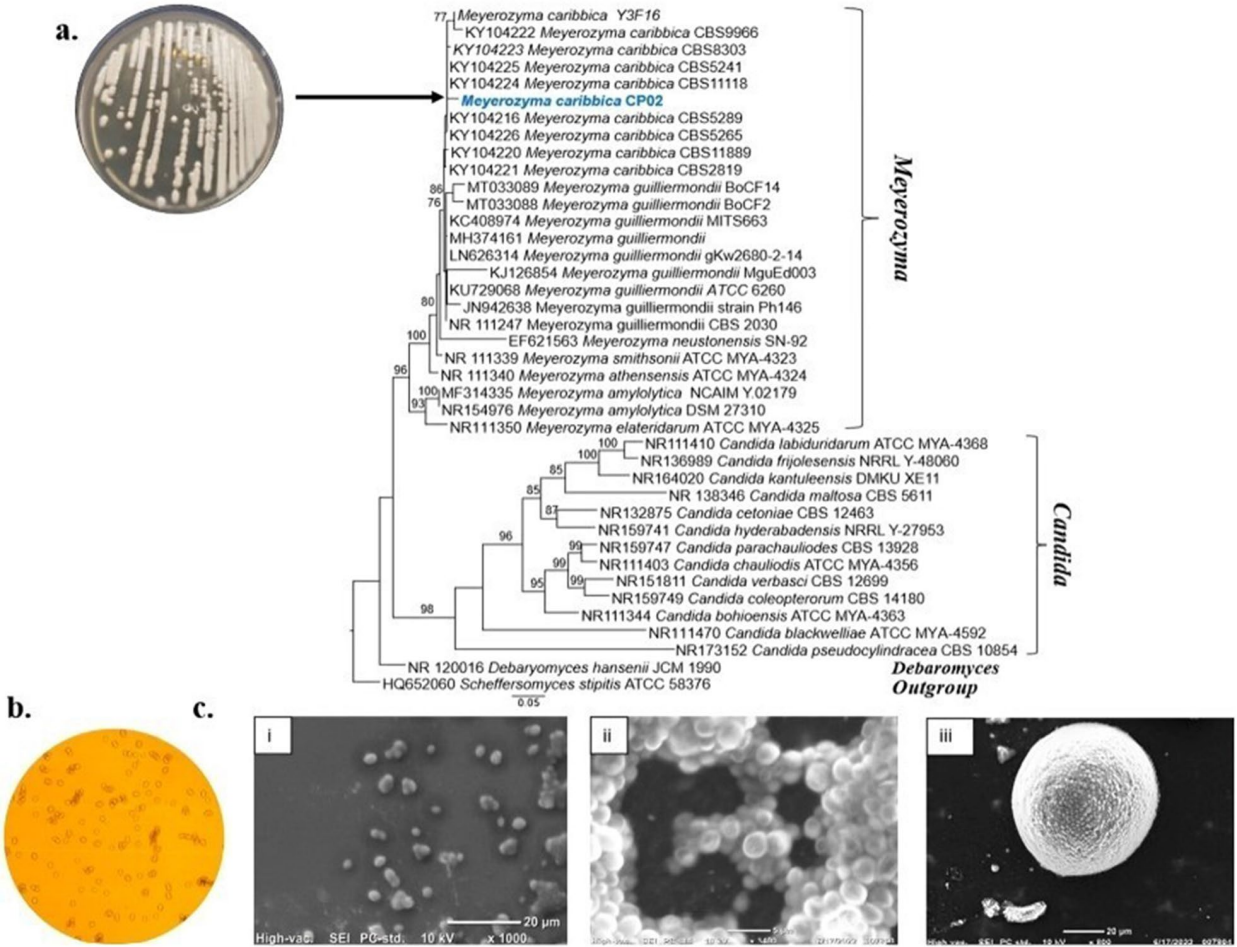
Isolation, screening and identification of the xylitol producing isolate. **a** Isolated strain CP02 on YPX plate along with its ITS and D1/D2 based phylogenetic tree showing the evolutionary relationship of *Meyerozyma caribbica* CP02 (ON077350) with other phylogenetically closest members. Bootstrap support values for ML ≥ 70% (black) given above the nodes. The tree has been rooted with the type strain of *Scheffersomyces stipites* ATCC58376. **b** Inverted microscope image of *M. caribbica* CP02 (magnification, 40×). **c** SEM micrograph depicting morphology and budding cells of *M. caribbica* CP02 at a magnification of, i. × 1000, yeast colony at ii. × 1100 and, iii. at × 800 with 10 kV

The isolate CP02 showed a desirable xylitol yield and therefore, it was selected for further study and identified using phylogenetic analysis. Based on Maximum Likelihood (ML) analysis of a combined dataset of the ITS and D1/D2 domain of 26S rDNA regions showed that our taxon clustered in a monophyletic clade with *Meyerozyma caribbica* strains, with 99.68% sequence similarity. Hence, the isolate CP02 was named by its scientific nomination plus the lab code, i.e., *Meyerozyma caribbica* CP02 and submitted under the GenBank accession number ON077350 (CP02). *M. caribbica* CP02 was characterized by an ovoid to elongate shape, 1.4 × 4—6 µm size, which either occurred singly, or in short chains. The colony appeared smooth, butyrous, glistening, and off-white in colour. Furthermore, *M. caribbica* has been shown to be a non-pathogenic, non-conventional, safe, and rapidly growing yeast even under microaerophilic conditions [35]. Additionally, *M. caribbica* has also been reported to possess antagonistic properties which maximizes the shelf life of food and beverages by inhibiting the growth of bacteria and fungi, therefore, making it an attractive candidate for a broad range of applications [36] (Fig. 1).

### Optimization of operational parameters affecting fermentation by ***M. caribbica*** CP02

#### Effect of temperature

**Fig. 2:**
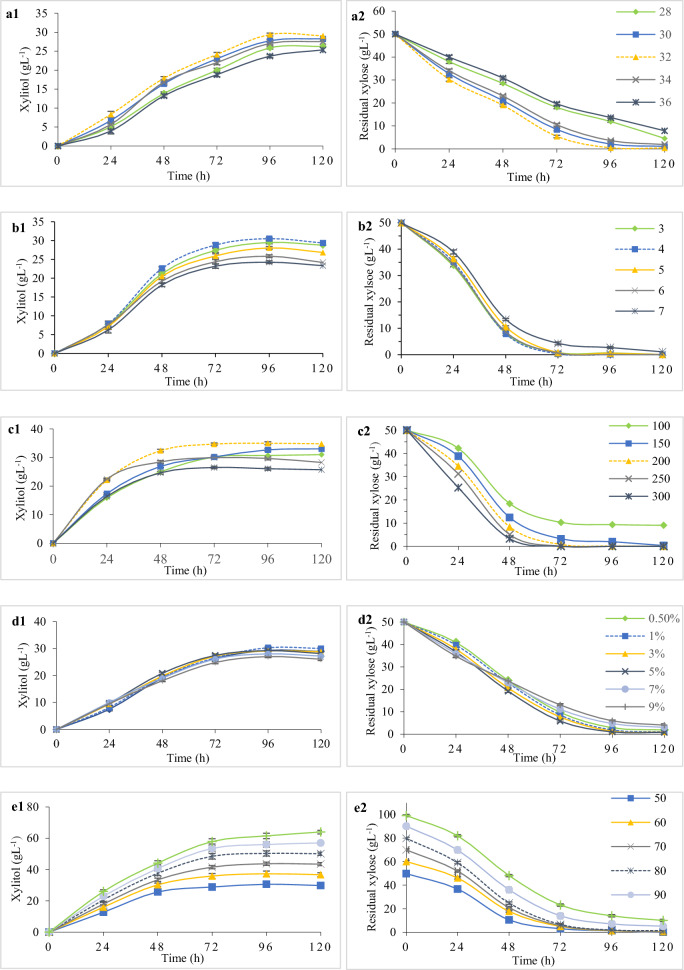
Optimization of process parameters. Time courses depicting the effect of different fermentation parameters such as, **a** Temperature (°C), **b** pH, **c** Agitation rate (rpm), **d** Inoculum size (% v/v) and, **e** Initial substrate (gL^−1^) on xylitol production (1) and xylose consumption (2)

A relatively stable range of xylitol titer from 27.06 ± 0.51 to 29.40 ± 0.62 gL^−1^ and productivity of 0.28 to 0.30 gL^−1^ h^−1^ was obtained in the temperature range of 30–34 °C (Fig. 2a). Though, the highest xylitol yield of 29.40 ± 0.64 gL^−1^ and productivity (Q_p_) of 0.30 gL^−1^ h^−1^ was observed at 32 °C. Temperature impacts microbial growth rate and xylitol production which might be the reason for almost 24% decrease in xylitol titer (23.40 ± 0.32 gL^−1^) at 36 °C as compared to xylitol titer at 32 °C. A subsequent reduction in xylose consumption by 27% was also observed as temperature also affects the regulation of transport proteins involved in xylose sequestration and enzyme activity [37].

#### Effect of pH

The experimental findings of a set of four flasks for batch xylitol bioconversion were studied to determine the effect of pH ranging from 3.5 to 7.5. Maximum xylitol production (30.5 ± 2.41 gL^−1^), yield (0.61 gg^−1^ xylose consumed), and productivity (0.32 gL^−1^ h^−1^) were observed at pH 4.0 after 96 h (Fig. 2b). It is possible that *M. caribbica* may have developed defenses against external pH fluctuations to preserve internal pH homeostasis which can be the reason behind, optimum production at acidic pH. Also, the enzymes involved in the metabolic pathway leading to xylitol production may perform best in moderately acidic environments since pH has a substantial impact on enzyme kinetics. Moreover, optimal xylitol production at low pH could be associated with a favorable redox balance, ensuring that the necessary reducing equivalents are available for efficient xylitol synthesis [33]. The xylitol yield ranged from 0.51gg^−1^ to 0.61gg^−1^ xylose consumed, indicating the activity of *M. caribbica* CP02 over a broad range of pH. In a similar study using corn cob hydrolysate, Nagarajan et al. [38] reported the optimum working pH of *M. caribbica* to be 3.69. *Candida tropicalis* produced 26.12 gL^−1^ xylitol in the optimum pH range of 3.0 to 4.0 at 200 rpm in a fermentation study on oil palm empty fruit bunch hydrolysate [39]. During second generation fermentation pH generally falls below 5.0 which heavily impacts cell viability. So, improving the tolerance to weak acids, is not just beneficial for improving the fermentation capacity, but also for offering competitive advantage over microbial contaminants which could contribute to observed high yield at low pH. Non-sterile fermentation is a major factor for economic consideration [40].

#### Effect of agitation rate

The amount of residual xylose in the fermented broth was 0.15 ± 0.02 gL^−1^ at 200 rpm, compared to 29.69 ± 0.19 gL^−1^ and 32.70 ± 0.22 gL^−1^ xylitol in flasks agitated at 250 and 150 rpm, respectively. However, xylose was completely consumed at 250 rpm, whereas 2.01 ± 0.05 gL^−1^ xylose remained unconsumed at 150 rpm during the same incubation period (96 h), as shown in Fig. 2c. The xylitol titer obtained at 100 rpm agitation was much lower (30.71 ± 0.41 gL^−1^) with 9.35 ± 0.11 gL^−1^ residual xylose. The lowest amount of xylitol was obtained at 300 rpm (26.08 ± 0.50 gL^−1^) with complete xylose consumption. This is consistent with the study of Prabhu et al. [35], which showed that the xylose utilization time decreased as agitation increased, with the maximum time taken at 120 rpm (96 h) and the shortest time taken at 300 rpm (72 h) for an initial xylose concentration of 60 ± 0.18 gL^−1^ using mutated *Pichia fermentans*. The xylitol titer also increased linearly up to 250 rpm, after which the yield dropped. The highest xylitol titer and yield obtained by them was 36.6 ± 0.41 gL^−1^ and 0.61 gg^−1^ respectively, at 250 rpm.

#### Effect of inoculum size

The effect of inoculum size on xylitol synthesis by *M. caribbica* CP02 was investigated at concentrations ranging from 0.5 to 9% (v/v). The highest xylitol production (30.30 ± 0.41 gL^−1^) was found at 1% (v/v) inoculum size, with approximately 98% xylose utilization after 96 h of incubation, as shown in Fig. 2d. Furthermore, when the size of the inoculum was increased from 1.0% (v/v) to 3.0 and 5.0% (v/v), the xylitol titer showed a gradual and insignificant decrease to 29.08 ± 0.32 and 29.20 ± 0.21 gL^−1^, with 98.2 and 98.4% consumption of the initial xylose, respectively. At 7% (v/v) and 9% (v/v) inoculum sizes, the minimum xylitol titer was 27.17 ± 0.38 gL^−1^ and 28 ± 0.50 gL^−1^, respectively, indicating that using a larger amount of seed culture resulted in the use of xylose for cell growth rather than xylitol production. Furthermore, using the aforementioned inoculum concentrations, only 90.58% and 88.12% of the xylose was utilized after 96 h. The decrease in xylose consumption could be since oxygen is essential for xylose uptake rate by pentose assimilating yeasts, and higher inoculum concentration reduces the oxygen level in the medium [41].

#### Effect of initial substrate

Flasks containing production media were formulated with xylose concentration varying from 50 to 100 gL^−1^ as depicted in Fig. 2e. The highest xylitol titer (50.3 ± 0.28 gL^−1^) with a specific xylitol yield of 0.64 gg^−1^, 0.52 gL^−1^ h^−1^ productivity, 1.87 ± 0.04 gL^−1^ residual xylose was recorded with the xylose amount of 80 ± 0.24 gL^−1^. In flasks containing initial xylose of 90 ± 0.71 gL^−1^ and 100 ± 1.1 gL^−1^, xylitol titer of 56 ± 0.21 gL^−1^ and 61.5 ± 0.28 gL^−1^ and a xylose consumption of 92.13% and 86.02% respectively, was recorded at 96 h. The xylitol titer further increased to 57 ± 0.30 gL^−1^ and 67 ± 0.35 gL^−1^ after 120 h of incubation with a yield of 0.67 gg^−1^ and 0.75 gg^−1^ xylose consumed respectively. This implies that increasing the concentration of initial substrate, increases the xylitol production, but at the expense of a longer incubation period as the yeast may take more time to metabolize and convert surplus xylose into xylitol. However, there is a limit to which how much substrate a microbe can effectively process and after a certain limit, substrate inhibition occurs which affects the microbial growth. Subsequently productivity drops as the yeast is unable to tolerate the elevated osmotic pressure due to increased solutes in the fermentation medium of a batch process [30]. Also, from the standpoint of economic feasibility, it may be desirable to shorten the fermentation time.

### Statistical validation

Statistical analysis is an effective way to identify and validate the significant factors affecting the yield of product. Analysis of variance (ANOVA) was employed to examine the studied experimental factors and their statistical significance in the performance of the recommended method.

The F-value (Fischer variance ratio) of the computed model was 122.15 and the p-value of < 0.0001, which implies p < 0.05 for regression model equation confirmed that the quadratic model fit well to the experimental results. The lack of fit value of 3.27 implied that the model’s variables significantly correlated with process response which was xylitol production [42]. Also, the difference between adjusted R^2^ (0.9838) and predicted R^2^ (0.9569) in the current study was less than 0.20, indicating that the model was reliable (Additional file 2: Table S2).

The regression equation for xylitol production in terms of coded factors, after a complete fermentation cycle as a function of the quadratic terms and associated interactions can be presented as:

Xylitol = 57.08 + 19.26A −0.0942B −0.7258C −0.1117D + 0.3250AB −1.72AC + 3.06AD −0.4000 BC + 3.27BD −3.97CD −9.38A^2^ -4.74B^2^ -2.32C^2^ -6.51D^2^

Where, A is initial xylose (gL^−1^), B is inoculum size (%), C is pH and D is agitation (rpm).

**Fig. 3:**
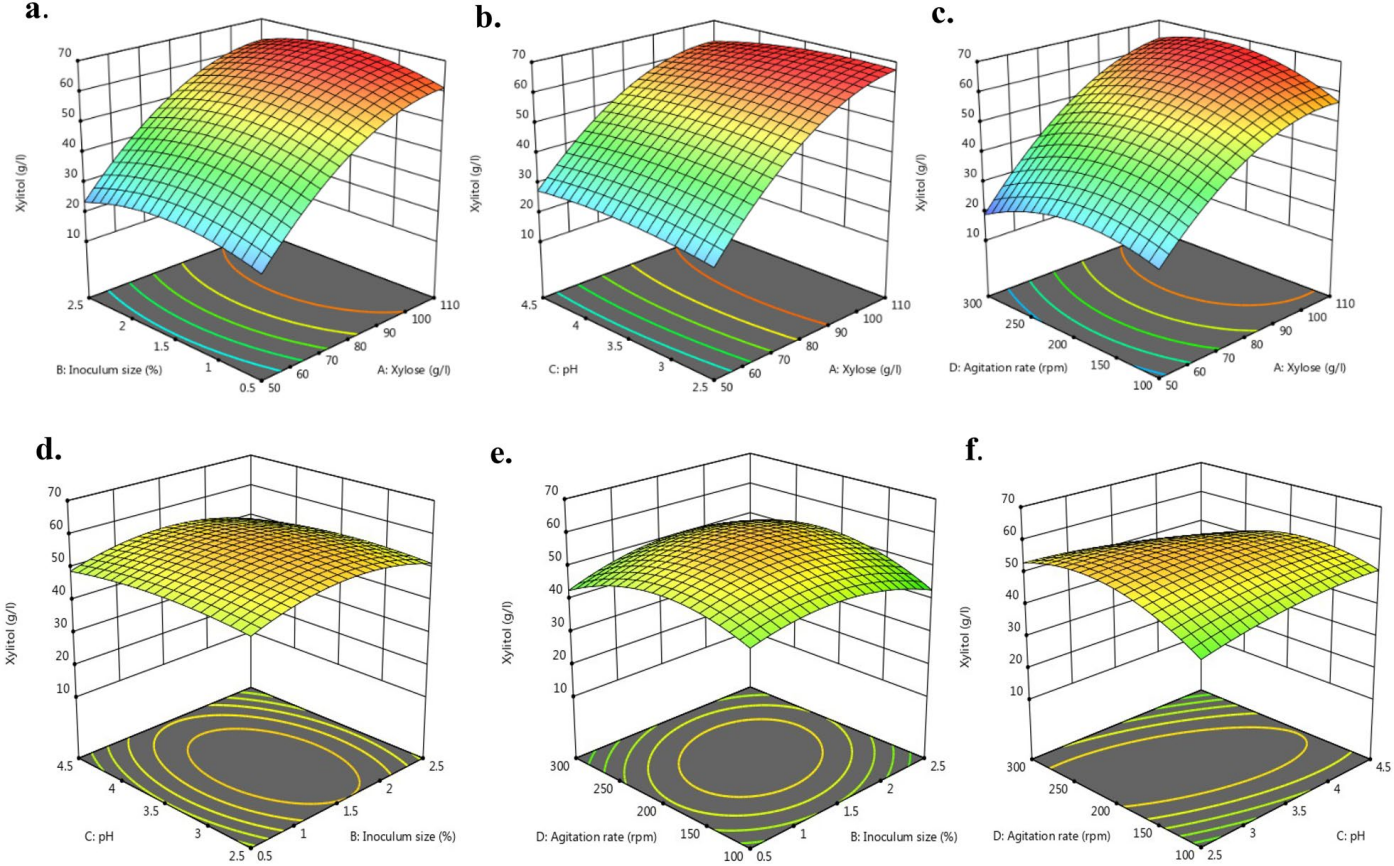
Response surface plots based on the polynomial model. The figure shows the interactive effects of, a. xylose (**A**) and inoculum size (**B**), b. xylose (**A**) and pH (C), c. xylose (**A**) and agitation rate (D), d. Inoculum size (**B**) and pH (C), e. Inoculum size (**B**) and agitation rate (**D**), f. pH (C) and agitation rate (D) on the production of xylitol (gL^−1^)

From the results, it was inferred that A, had a positive influence on the production of xylitol as compared to other variables under study. In contrast to the effect of other factors, where xylitol production grew with increasing factor values up to a certain point and dropped beyond that, it was deduced that xylitol production increased with an increase in starting xylose concentration. Likewise, interaction of AB, AD and BD also affected the xylitol production positively. Variables with higher F values and lower p values are more significant [43]. The independent model variables (p < 0.05) having a significant effect on response, were determined from ANOVA table provided in Additional file 2: Table S3. 3D interaction plots between the two experimental variables have been presented in Fig. 3. Factor A (initial xylose concentration) was the most significant factor (p < 0.0001) as can be seen in Fig. 3a, b, and c, where xylitol concentration increased sharply irrespective of the other factor under observation. In a similar study, Yewale et al. [44], conducted central composite design (CCD) and found that xylose concentration was one of the major factors for xylitol production. Figure 3a shows the response surface plot showing the interactive effect of (AB) of initial xylose concentration (A) and inoculum size (B) on xylitol titer (gL^−1^) which validates that the xylitol yield (gL^−1^) rises with increase in xylose titer from 50 to 110 gL^−1^ for inoculum size between 0.5 to 2.5% (v/v). The influence of AC (xylose and pH) on the response revealed that the xylitol titer (gL^−1^) increased as the initial xylose concentration of the fermentation media approached from 50 to 110 gL^−1^ irrespective of the changes in initial pH from 2.5 to 4.5 (Fig. 3b). Furthermore, the interactive effect of BD (inoculum size and agitation rate) demonstrated that the xylitol yield enhanced as the inoculum size increased from 0.5 to 2.5% (v/v) for the studied rate of agitation from 100 to 300 rpm (Fig. 3c). As can be deduced from the surface plots agitation rate appears to be the second most significant factor after xylose, (Fig. 3e) and interaction of B (inoculum size, %) and D (agitation rate) has a positive influence on xylitol production, followed by BC (Fig. 3f). Interaction of BC (Fig. 3d) was the least significant (p > 0.6586) as far xylitol accumulation is concerned (Additional file 2: Table S3). The desired goal for each operational condition (initial xylose concentration (gL^−1^), inoculum size (%), pH, agitation (rpm), and response (xylitol, gL^−1^), according to the software optimization step, was defined as "within" the range in order to generate the best conditions that are also feasible for the fermentation of hydrolysate-based medium. For instance, in a bid to elevate the initial concentration of xylose in rice straw hydrolysate, inhibitors may accumulate to a degree that is detrimental to the growth of yeast. After combining each desirability into a single value, the software looks for ways to optimize this function in relation to the response goal. As can be shown in Additional file 2: Table S3, under ideal operating conditions (xylose concentration of 80 g/l, inoculum % of 1.5, pH 3.5 and agitation rate 200 rpm), the model predicts 57.08 gL^−1^ xylitol. Under these ideal circumstances, the value of the desirability function was discovered to be 1.0. An additional test was then performed and the projected reaction value and the lab experiment agree quite well.

### Influence of variation of agitation speed on xylitol production

A batch fermentation with sequential two stage agitation pattern, which involved altering the agitation speed at a time duration of 34 h (optimized previously), was efficient for enhancing the xylitol yield.

The highest agitation level, 250/200 rpm (250 rpm in the first stage for 34 h and 200 rpm in the second stage), resulted in the highest cell biomass (22.83 ± 0.14 gL^−1^) with the lowest xylitol yield (0.64 ± 0.23 gg^−1^). The lowest shaking speed level of 150/100 rpm resulted in the lowest cell biomass concentration (11.20 ± 0.08 gL^−1^), xylitol titer (53.93 ± 0.28 gL^−1^) and a moderately high xylitol yield (0.74 gg^−1^) with a maximum residual xylose concentration of 7.06 ± 0.12 gL^−1^ as shown in Additional file 2: Table S5.

**Fig. 4:**
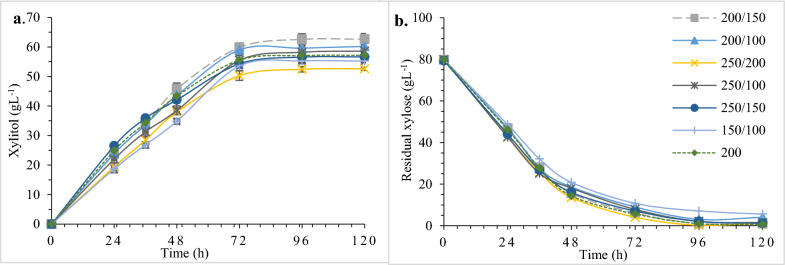
Study of two step agitation approach on fermentation by *M. caribbica* CP02. Depicting the trend of, **a** xylitol production and, **b** xylose consumption at different combinations of shaking speed

However, agitation at 200/150 rpm showed the highest xylitol production (61.10 ± 0.51 gL^−1^), xylitol yield (0.77 gg^−1^), xylose to xylitol bioconversion efficiency (84.61%), the best xylitol productivity (0.64 gL^−1^ h^−1^) and complete consumption of xylose at 96 h (refer Fig. 4a, b) as compared to control which yielded 57.10 ± 0.45 gL^−1^ xylitol, 0.72 xylitol gg^−1^ xylose consumed and 0.59 gL^−1^ h^−1^ productivity. This trend can be supported by the fact that xylose metabolism in the cells is mainly governed by the presence of key enzymes xylose reductase (XR) and xylitol dehydrogenase (XDH) and, and their co-factors (NADPH and NAD +) respectively. Under excess oxygen supply, NADH produced during xylitol to xylulose conversion is re-oxidized by the respiratory chain, and xylitol is consumed for the cell growth. On the contrary, in the presence of limited oxygen, xylose metabolic pathway is affected as redox imbalance occurs between NAD + and NADH. This results in the over accumulation of NADH, subsequently inhibiting XDH and promoting xylitol buildup [45]. Zhang et al. [30] used *C. athensensis* SB18 and reported that the agitation at 200 rpm for first 24 h followed by 100 rpm for rest of the incubation period exhibited the maximum xylitol titer (115.62 gL^−1^) from 150 gL^−1^ initial xylose and xylitol yield (0.77 gg^−1^) at 30 °C for 108 h. Another study using corn cob hydrolysate found that increasing the aeration rate from 1.5 vvm to 3 vvm after 24 h resulted in higher xylitol yield (0.76 gg^−1^) [11]. In a slightly different approach, Dasgupta et al. [46] studied the fermentability of corncob hydrolysate using *P. caribbica* in a 5 L stirred tank bioreactor in a repeated fed- batch mode, run initially at 28 °C, pH 6.0, 300 rpm, 1.5 vvm aeration followed by cell harvestation upon ~ 90% xylose consumption. Next, conditions were set at 170 rpm and 0.5 vvm aeration for microaerobic conditions and fresh media supplementation. After a couple of batches, the microbe produced 124.1 ± 0.45 g/L xylitol and yield of 0.80 ± 0.02 g/g. Therefore, two stage agitation approach is ideal for getting maximum xylitol yield, by developing a microaerobic environment as a balance between cell development and xylitol accumulation [45].

### Composition of RS hemicellulosic hydrolysate

Pretreated and detoxified RSH was vacuum concentrated to obtain 100 ml of RSH fractions, RSH1, RSH2 and RSH3 with initial xylose concentrations of 59.22 ± 1.0, 78.62 ± 0.71 and 98.73 ± 0.93 gL^−1^ respectively. The major components of 1.5% v/v H_2_SO_4_ catalyzed untreated hydrolysate were (gL^−1^): sugars (1.0 ± 0.26 glucose, 14.91 ± 0.53 xylose, and 3.60 ± 0.79 arabinose), aliphatic acids (1.5 ± 0.13 acetic acid, 0.28 ± 0.08 formic acid, and 0.04 ± 0.01 levulinic acid), furans (0.029 ± 0.017 HMF and 0.112 ± 0.002 furfural) and total phenols (0.98 ± 0.007). As previously reported, there was a significant decrease in the amount of these inhibitors after detoxification and evaporation due to vacuum concentration [31]. However, inhibitors began to accumulate again as RSH concentration increased, with RSH1 having the least amount of total organic acids (1.54 ± 0.10 gL^−1^), furans (0.049 ± 0.004 gL^−1^), total phenols (0.64 ± 0.26 gL^−1^) and RSH3 having the most, i.e., 2.43 ± 0.25 gL^−1^, 0.078 ± 0.061 gL^−1^ and 2.47 ± 0.052 gL^−1^, respectively. Similarly, as shown in Table 1, the increase in initial xylose concentration was accompanied by an increase in the amount of glucose, arabinose, and inhibitors as well.

### Study of xylitol production by *M. caribbica* CP02 with RSH

**Fig. 5:**
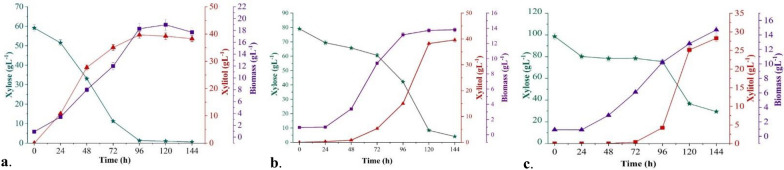
*M. caribbica* CP02 led shake flask fermentation. Time course depicting the trend of xylose consumption ( ), xylitol production ( ) and, biomass generation ( ) from **a** RSHM1, **b**. RSHM2 and, **c** RSHM3

Two stage agitation during fermentation was carried out in 500 mL shake flasks in batch mode using RSH derived media (RSHM) at optimized conditions to assess the RSH fermentability of *M. caribbica* CP02 under high initial xylose concentrations as compiled in Table 2. As can be seen, the xylitol titer decreased as the level of RSH concentration increased. RSHM1 had the highest xylose titer (36.10 ± 0.40 gL^−1^), xylitol yield (0.62 gg^−1^), and productivity (0.38 gL^−1^ h^−1^) (Fig. 5a). In comparison to the 500 mL shake flask model fermentation with a similar amount of commercial xylose (80 gL^−1^) taken initially under the same conditions, a reduction of nearly 75% of xylitol titer (15.01 gL^−1^) and 48% of yield (0.44 gg^−1^) was observed after 96 h incubation (Sect. "Composition of RS hemicellulosic hydrolysate"). A similar study using *C. tropicalis* GS18 produced a xylitol yield of 0.60 gg^−1^ and a xylitol titer of 34.21 gL^−1^ from detoxified RS hydrolysate concentrated to obtain an initial xylose level of 58.78 gL^−1^ [47]. The accumulated biomass dry weight decreased from 18.90 ± 0.78 gL^−1^ (RSHM1) to 13.03 ± 0.91 gL^−1^ (RSHM2) and 9.96 ± 0.90 gL^−1^ (RSHM3). This decrease could be attributed to the combined stress exerted by elevated levels of total solids and inhibitors at highly concentrated RSH. Furans and their corresponding alcohols have been shown to reduce cell biomass yield, whereas aliphatic acids inhibit cell growth by facilitating the influx of dissociated form, thereby increasing H^+^ ions into the cytoplasm [48]. Moreover, lag phase of *M. caribbica* CP02 stretched from about 12 h in RSHM1 to 48 h and 72 h in RSHM2 (Fig. 5b) and RSHM3 (Fig. 5c) respectively. Although, a sudden increase in xylitol production (38.22 ± 0.72 gL^−1^) was observed at the beginning of stationary phase i.e., 120 h on RSHM2, indicating the good adaptability, tolerance, and fermentation efficiency of *M. caribbica* CP02 even with high initial xylose concentration (78.62 ± 0.71 gL^−1^) and inhibitors such as acetic acid (2.06 ± 0.16 gL^−1^), formic acid (0.11 ± 0.07 gL^−1^), levulinic acid (0.03 ± 0.02 gL^−1^), 5-HMF (0.018 ± 0.001 gL^−1^) and furfural (0.044 ± 0.002 gL^−1^). This implies that *M. caribbica* CP02 could produce good xylitol yields in RS-derived media with initial xylose concentrations as high as 80 gL^−1^ but, at the expense of a longer process time. A reduction in sugar consumption and partial inhibition was observed when the initial xylose concentration in RS hydrolysate was further increased to 98.73 ± 0.93 gL^−1^. In this case at 96 h, the maximum xylitol titer obtained was only 4.24 ± 0.59 gL^−1^ as compared to the amount of xylose consumed (23.27 ± 0.52 gL^−1^). However, the xylitol titer rose to 25.15 ± 0.34 gL^−1^ by the end of 120 h. This pattern can be attributed to the fact that under adverse conditions a large amount of xylose is consumed by the catabolic reaction through the TCA cycle and for biomass production, and only a minor amount of substrate is employed for xylitol formation. Similar observation was made by Sampaio et al., [34] while studying the effect of temperature on the xylitol production efficiency of *Debaryomyces hansenii* UFV-170. The study reported that at low temperature (15 °C), almost 46% and 43% of the initial xylose was involved in catabolic reactions and biomass production, only 11% of the substrate was used for xylitol formation. The decrease in xylose to xylitol bioconversion efficiency of *M. caribbica* CP02 could also be accredited to an increase in RSHM viscosity due to an increase in the amount of dry matter with volume reduction of RSH to achieve the desired xylose concentration. High viscosity might have influenced yeast metabolic activity and fermentation kinetics [49]. A study focused majorly on ethanol production and partially on xylitol on *M. caribbica* URM 8365 with biomass hydrolysate also reported that at condition of 1.6 gL^−1^ acetic acid in the medium, only xylose metabolism was hindered while the uptake of glucose and total ethanol production remained unaffected [6]. RSHM1 was chosen for further research after considering all the factors influencing the feasibility of the fermentation process, such as incubation time, nutrient consumption, and total inhibitor accumulation.

**Table 2:** Shake flask study summarizing fermentation variables of xylitol production by *M. caribbica* CP02 using RSHM

	Residual xylose (gL^−1^)	Xylitol^#^ (gL^−1^)	X (gL^−1^)	Y_P/S_ (gg^−1^)	Y_P/X_ (gg^−1^)	Qp (gL^−1^ h^−1^)	η (%)
RSHM* 1	01.28 ± 0.31^c^	36.10 ± 0.40^a^	18.90 ± 0.78^a^	0.62	1.91	0.38	68.88
RSHM 2	42.17 ± 1.07^b^	15.01 ± 0.36^b^	13.03 ± 0.91^b^	0.40	1.15	0.16	44.44
RSHM 3	75.46 ± 0.50^a^	4.24 ± 0.59^c^	9.96 ± 0.90^c^	0.18	0.43	0.04	20.00

*, Rice straw hydrolysate derived media; ^#^, Xylitol titer after 96 h of fermentation

X: biomass, Y_P/S_: yield (g xylitol) (g xylose consumed)^−1^, Y_P/S_: yield (g xylitol) (g biomass produced)^−1^, Qp: xylitol productivity, η: xylose to xylitol bioconversion efficiency

Values with different letters in the same column showed significant differences (P ≤ 0.05)

### RSH based two-stage agitation and aeration fermentation in a 3L batch bioreactor

**Fig. 6:**
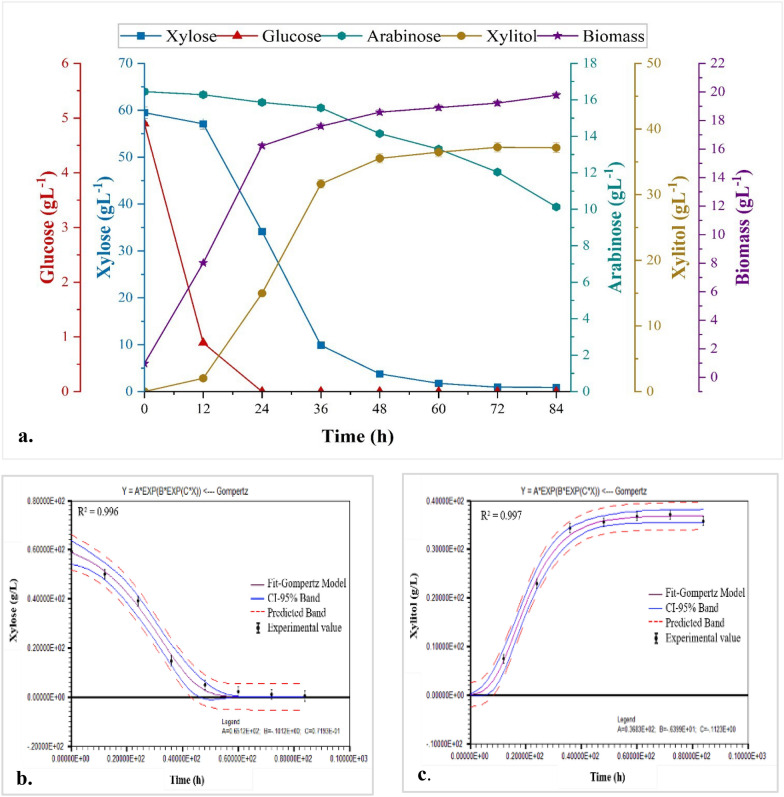
3-L batch bioreactor study. **a** Time course for two step agitation and aeration approach during fermentation, depicting the profiles of xylose assimilation, xylitol production, and biomass formation by *M. caribbica* CP02 from RSHM1. Kinetic profiles of **b**. xylose consumption and **c** xylitol formation by Gompertz model

The two-stage agitation and aeration strategy during fermentation followed the process of Shue et al. [50], with modifications aimed at desirable level of xylitol production. As shown in Fig. 6a, it took 72 h to consume 59.48 ± 0.82 gL^−1^ xylose, whereas glucose was utilized by the end of 24 h and arabinose was partially metabolized, but at a slow rate with no arabitol formation. Although, arabinose being the second major monosaccharide after xylose did not affect xylose utilization and xylitol production adversely. Furthermore, arabinose assimilation began only after xylose was depleted. These observations are supported by the findings of Villarreal et al. [51], who discovered that most yeast strains, including *Candida* sp., are unable to or only partially use arabinose. Furthermore, the cell biomass continued to increase even after the consumption of glucose, indicating that the growth of biomass after 12 h was primarily attributed to the consumption of xylose. Similar results were reported by Saha and Kennedy [52], who used *Barnettozyma populi* NRRL Y-12728 to produce xylitol from corn stover hydrolysate. They discovered that *B. populi* Y-12728 could produce 19.7 gL^−1^ xylitol, 0.15 gL^−1^ h^−1^ productivity, and 0.51 gg^−1^ xylose consumed in 135 h from 150 gL^−1^ xylose.

58.64 ± 0.39 gL^−1^ of xylose was consumed to produce maximum xylitol titer of 37.20 ± 0.48 gL^−1^ and a bioconversion efficiency of 70%. A xylitol yield of 0.63 gg^−1^ and, productivity of 0.52 gL^−1^ h^−1^ was obtained in batch bioreactor as compared to 0.38 g L^−1^ h^−1^ productivity obtained in the 500-mL shake flask previously, with a similar initial xylose concentration using RSHM. The improvement in xylitol productivity might be due to the improved mixing phenomena and higher mass transport efficiency achieved in the fermenter growth vessel. Moreover, analysis through the Gompertz Model kinetics showed a good fit, implying that both xylose consumption and xylitol formation aligned closely with the experimental data (Fig. 6b, c). Furthermore, in a 500-mL shake flask study, even though the rate of biomass growth decreased during the xylitol accumulation stage, cell growth continued to reach its maximum at 60 h. In bioreactor fermentation, on the other hand, the biomass concentration peaked at 48 h and the final cell dry weight was 19.22 ± 0.64 gL^−1^. The rapid biomass growth during the cell growth phase can be linked to a faster xylitol accumulation rate and, as a result, improved productivity. This is in correspondence with the findings of Silva et al. [53], in a study conducted on *C. guilliermondii* using rice straw hydrolysate as a substrate. The work reported a similar increase in xylitol productivity when switching from aerated flasks (agitation, 250 rpm) to 1L batch bioreactor set at 550 rpm and 0.4 vvm. The yield reported in this study is more than that reported by Kaur et al. [47] on RS hydrolysate (initial xylose, 51.57 gL^−1^) by adapted strain of *M. caribbica* which produced xylitol titer of 29.95 gL^−1^, yield of 0.58 gg^−1^ and productivity of 0.42 gL^−1^ h^−1^). Using *C. guilliermondii*, the researchers reported a xylitol yield, volumetric productivity, and efficiency of 0.59 gg^−1^, 0.54 gl^−1^ h^−1^, and 64.3%, respectively, from RSH with an initial xylose concentration of 81.4 gL^−1^. Similarly, a 1L bioreactor study on sugarcane bagasse hydrolysate using *M. caribbica* JA9 with 40 gL^−1^ xylose yielded 0.54 gg^−1^, which was found to be greater than the control strain *M. guilliermondii* (0.44 gg^−1^) under the same conditions [54].

## Conclusion

Bioprospecting lignocellulosic agricultural waste and selective enrichment on RS derived xylose-rich medium resulted in the isolation and purification of *M. caribbica* CP02, a new xylitol producing yeast. *M. caribbica* CP02's tolerance to extreme acidic conditions reduces the risk of bacterial contamination during fermentation. In shake flask experiments, commercial xylose yielded 0.74 gg^−1^ (η, 86.81%) and RSH-based media yielded 0.64 gg^−1^ (η, 70.32%) xylitol, respectively. On RSH media, a 3-L bioreactor study of *M. caribbica* CP02 produced a high xylitol titer of 37.13 gL^−1^ with a xylitol yield of 0.63 gg^−1^, which is the highest in the literature from rice straw hydrolysate. The present study suggests that *M. caribbica* CP02 demonstrates promise for further scaling up and potential use as a robust yeast strain in the commercial production of xylitol. Additionally, the experimental data could be useful for processes where xylitol is sought as an alternative by-product in integrated biorefineries aiming for holistic biomass utilization.

### Supplementary Information

**Additional file 1.** Media standardization.

**Additional file 2.** Supplementary tables.

## Data Availability

Available on reasonable request.

## Abbreviations

ANOVA: Analysis of variance
HMF: Hydroxymethylfurfural
ITS: Internal transcribed spacer
OVAT: One variable at a time
RS: Rice straw
RSH: Rice straw hydrolysate
RSM: Response surface methodology
RSHM: Rice straw hydrolysate media
vvm: Volume of air/volume of media
