# Supplementary material for: Bioprocess optimization for enhanced xylitol synthesis by new isolate Meyerozyma caribbica CP02 using rice straw
Source: Biotechnol Biofuels Bioprod. 2024 Feb 24;17:31. doi: 10.1186/s13068-024-02475-8 (PMC10894501; doi:10.1186/s13068-024-02475-8)
Supplement: Supplementary file 1 — Additional file 1. Media standardization. [file 13068_2024_2475_MOESM1_ESM.docx]

**Media standardization**

**1. Effect of media components on xylitol production**

The influence of the nitrogen sources was studied by supplementing several nitrogen sources (1% w/v) such as ammonium sulphate, NH_3_(SO_4_)_2_; sodium nitrate, Na(NO)_3_; urea, CO(NH_2_)_2_ and peptone to the basic production media comprising (g/l): 5.0 xylose; 10 yeast extract; 10 peptone; 1 MgSO_4_. 7H_2_O; 0.5 CaCl_2_.7H_2_O and 0.5 KH_2_PO_4_, pH 5.5 at 32°C, 200 rpm for 96 h (Goli et al., 2020). The same production media without any supplementation was used as control. To further evaluate the effect of media components on xylose assimilation and xylitol formation, the following five media were prepared and further evaluated for their suitability or alteration to the current production media, designated as (g/l): M1 (7.5 yeast extract), M2 (3.0 Yeast extract, 5.0 Malt extract), M3 (5.0 NH_3_(SO_4_)_2_, 3.0 Yeast extract), M4 (3.0 yeast extract, 3.0 malt extract, 5.0 peptone), M5 (6.75 yeast extract, 2.0 malt extract) at pH 5.5. The defined mineral medium contained, (g/l): 0.5 CaCl_2_, 1 MgSO_4_. 7H_2_O, 0.5 KH_2_PO_4_, and 5 D-xylose (Trichez et al., 2019).

- 1. **Effect of nitrogen sources on xylitol production**

At 96 h maximum xylitol titer was obtained and the results are presented in **Fig. 1SF(a)**. It was noticed that yeast extract was the most significant nitrogen source (media formulation, M1) for yeast strain CP02 mediated xylitol synthesis with the maximum xylitol titer of 31.6 gL^-1^ and 0.36 gL^-1^ residual xylose (not shown) at 96 h in fermentation media. This corresponds to the maximum xylitol yield of 0.64 gg^-1^ and a xylitol productivity of 0.33 gL^-1^ h^-1^ as compared to the control with 22.05 gL^-1^ xylitol, 0.47 gg^-1^ yield and 3.13 gL^-1^ residual xylose. The minimal nitrogen requirement is in line to what is reported in the literature which supports the fact that low carbon to nitrogen ratio, favors cell growth whereas, metabolite formation is favored by high carbon to nitrogen ratio (Rodrigues et al., 2006).

**1.2 Effect of media components**

M1 showed the maximum xylitol titer of 33.75 gL^-1^ after 96 h, residual xylose, 1.12 gL^-1^ and a xylitol yield of 0.69 gg^-1^ xylose consumed and a productivity of 0.40 gL^-1^h^-1^ (**Fig. 1SF(b)**). Xylitol titer started decreasing gradually after 120 h, perhaps due to the further conversion of xylitol to xylulose (Trichez et al., 2019). Evidently, M1 was better than the other 4 media in relation to xylose consumption, maximum xylitol production and productivity, also signifying the essentiality of yeast extract with respect to xylitol fermentation.

**S1(b)**

**Fig 1SF.** Time courses of xylitol production and xylose (50 gL^-1^) consumption by *M. caribbica* CP02 depicting, 1SF(a). effect of nitrogen sources and 1SF(b), effect of different media composition at 32 $^{\circ}$C and 200 rpm.

**References**

Goli, JK., Panda, S.H., Linga, V.R., Bee, H., 2020. Statistical optimization of fermentation parameters using placket-burman for enhanced xylitol production by *Pichia stipitis* NCIM 3498. Plant Archives 20, 4447-4454.

Rodrigues, R.C.L.B., Sene L., Matos G.S., Roberto I.C., Pessoa Jr. A., Felipe MGA., 2006. Enhanced xylitol production by precultivation of *Candida guilliermondii* cells sugarcane bagasse hemicellulosic hydrolysate. Curr. Microbiol. 53, 53-59. [10.1007/s00284-005-0242-4](https://doi.org/10.1007/s00284-005-0242-4)

Trichez, D., Steindorff, A.S., Soares, C.E.V.F., Formighieri, E.F., Almeida, J.R.M., 2019. Physiological and comparative genomic analysis of new isolated yeasts *Spathaspora* sp. JA1 and *Meyerozyma caribbica* JA9 reveal insights into xylitol production. FEMS Yeast Research, 19: foz034.  10.1093/femsyr/foz034
