# Supplementary material for: Bioprocess optimization for enhanced xylitol synthesis by new isolate Meyerozyma caribbica CP02 using rice straw
Source: Biotechnol Biofuels Bioprod. 2024 Feb 24;17:31. doi: 10.1186/s13068-024-02475-8 (PMC10894501; doi:10.1186/s13068-024-02475-8)
Supplement: Supplementary file 2 — Additional file 2. Supplementary tables. [file 13068_2024_2475_MOESM2_ESM.docx]

**Supplementary tables**

**Table 1T** Set of designed experiments

|  |  | Factor 1 | Factor 2 | Factor 3 | Factor 4 | Response 1 |
| --- | --- | --- | --- | --- | --- | --- |
| Std | Run | A: Xylose | B: Inoculum size | C: pH | D: Agitation rate | Xylitol |
|  |  | g/l | % |  | rpm | g/l |
| 19 | 1 | 50 | 1.5 | 4.5 | 200 | 27.99 |
| 7 | 2 | 80 | 1.5 | 2.5 | 300 | 53.7 |
| 6 | 3 | 80 | 1.5 | 4.5 | 100 | 51.46 |
| 5 | 4 | 80 | 1.5 | 2.5 | 100 | 45.8 |
| 11 | 5 | 50 | 1.5 | 3.5 | 300 | 18.26 |
| 3 | 6 | 50 | 2.5 | 3.5 | 200 | 23.9 |
| 27 | 7 | 80 | 1.5 | 3.5 | 200 | 56.42 |
| 23 | 8 | 80 | 0.5 | 3.5 | 300 | 40.26 |
| 22 | 9 | 80 | 2.5 | 3.5 | 100 | 43.16 |
| 18 | 10 | 110 | 1.5 | 2.5 | 200 | 64.52 |
| 13 | 11 | 80 | 0.5 | 2.5 | 200 | 52.3 |
| 2 | 12 | 110 | 0.5 | 3.5 | 200 | 62.08 |
| 15 | 13 | 80 | 0.5 | 4.5 | 200 | 49.71 |
| 8 | 14 | 80 | 1.5 | 4.5 | 300 | 43.47 |
| 20 | 15 | 110 | 1.5 | 4.5 | 200 | 62.39 |
| 17 | 16 | 50 | 1.5 | 2.5 | 200 | 23.22 |
| 26 | 17 | 80 | 1.5 | 3.5 | 200 | 57.14 |
| 21 | 18 | 80 | 0.5 | 3.5 | 100 | 42.75 |
| 9 | 19 | 50 | 1.5 | 3.5 | 100 | 24.06 |
| 14 | 20 | 80 | 2.5 | 2.5 | 200 | 52.13 |
| 29 | 21 | 80 | 1.5 | 3.5 | 200 | 58.95 |
| 1 | 22 | 50 | 0.5 | 3.5 | 200 | 26.09 |
| 10 | 23 | 110 | 1.5 | 3.5 | 100 | 58.98 |
| 28 | 24 | 80 | 1.5 | 3.5 | 200 | 56.6 |
| 4 | 25 | 110 | 2.5 | 3.5 | 200 | 61.19 |
| 16 | 26 | 80 | 2.5 | 4.5 | 200 | 47.94 |
| 25 | 27 | 80 | 1.5 | 3.5 | 200 | 56.29 |
| 12 | 28 | 110 | 1.5 | 3.5 | 300 | 65.44 |
| 24 | 29 | 80 | 2.5 | 3.5 | 300 | 48.74 |

**Table 2T Model fit summary**

| **Source** | **Sum of squares (SS)** | **Degree of freedom** | **Mean square** | **F-value** | **p-value** |
| --- | --- | --- | --- | --- | --- |
| Regression | 5370.10 | 14 | 383.58 | 122.15 | <0.0001 |
| Residual error | 43.96 | 14 | 3.14 |  |  |
| Lack of fit | 39.17 | 10 | 3.92 | 3.27 | 0.1323 |
| Pure error | 4.79 | 4 | 1.20 |  |  |
| Total error | 5414.06 | 28 |  |  |  |
| R^2^= 0.9871; Predicted R^2^= 0.9296; Adjusted R^2^ = 0.9743;  Adeq. precision = 30.2049 | | | | | |

**Table 3T** ANOVA table for quadratic model

| **Source** | **Sum of Squares** | **df** | **Mean Square** | **F-value** | **p-value** |  |
| --- | --- | --- | --- | --- | --- | --- |
| **Model** | 5370.10 | 14 | 383.58 | 122.15 | < 0.0001 | significant |
| A-Xylose | 4449.83 | 1 | 4449.83 | 1417.04 | < 0.0001 |  |
| B-Inoculum size | 0.1064 | 1 | 0.1064 | 0.0339 | 0.8566 |  |
| C-pH | 6.32 | 1 | 6.32 | 2.01 | 0.1778 |  |
| D-Agitation rate | 0.1496 | 1 | 0.1496 | 0.0477 | 0.8304 |  |
| AB | 0.4225 | 1 | 0.4225 | 0.1345 | 0.7193 |  |
| AC | 11.90 | 1 | 11.90 | 3.79 | 0.0719 |  |
| AD | 37.58 | 1 | 37.58 | 11.97 | 0.0038 |  |
| BC | 0.6400 | 1 | 0.6400 | 0.2038 | 0.6586 |  |
| BD | 42.71 | 1 | 42.71 | 13.60 | 0.0024 |  |
| CD | 63.12 | 1 | 63.12 | 20.10 | 0.0005 |  |
| A² | 570.81 | 1 | 570.81 | 181.77 | < 0.0001 |  |
| B² | 145.71 | 1 | 145.71 | 46.40 | < 0.0001 |  |
| C² | 34.83 | 1 | 34.83 | 11.09 | 0.0050 |  |
| D² | 274.97 | 1 | 274.97 | 87.56 | < 0.0001 |  |
| **Residual** | 43.96 | 14 | 3.14 |  |  |  |
| Lack of Fit | 39.17 | 10 | 3.92 | 3.27 | 0.1323 | not significant |
| Pure Error | 4.79 | 4 | 1.20 |  |  |  |
| **Cor Total** | 5414.06 | 28 |  |  |  |  |

**Table 4T** Optimization results for xylitol

| No. | Xylose, g/l | Inoculum size, % | pH | Agitation, RPM | Xylitol,g/l | Desirability |
| --- | --- | --- | --- | --- | --- | --- |
| 1 | 80 | 1.5 | 3.5 | 200 | 57.08 | 1.0 |
| Lab experiment | | | | | 56.85±0.44 |  |

**Table 5T.** Summary of fermentation variables under two step agitation

| **Agitation (rpm)** | **Xylitol (gL^-1^)** | **Residual xylose (gL^-1^)** | **Biomass, X (gL^-1^)** | **Yield, Y_P/S_ (gg^-1^)** | **Productivity, Qp (gL^-1^h^-1^)** | **Conversion efficiency, ⴄ (%)** |
| --- | --- | --- | --- | --- | --- | --- |
| 200 | 57.10 | 0.87 | 17.51 | 0.72 | 0.59 | 79.12 |
| 200/150 | 61.10 | 1.01 | 20.13 | 0.77 | 0.64 | 84.61 |
| 200/100 | 58.04 | 3.00 | 16.7 | 0.75 | 0.60 | 82.41 |
| 250/200 | 51.10 | 0.19 | 22.83 | 0.64 | 0.53 | 70.32 |
| 250/100 | 56.87 | 2.05 | 17.67 | 0.73 | 0.60 | 80.21 |
| 250/150 | 55.22 | 1.97 | 19.02 | 0.70 | 0.58 | 76.92 |
| 150/100 | 53.93 | 7.06 | 11.20 | 0.74 | 0.56 | 81.31 |
